# Role of inflammasome regulation on immune modulators

**DOI:** 10.7555/JBR.32.20170120

**Published:** 2018-02-12

**Authors:** Huijeong Ahn, Hyuk Moo Kwon, Eunsong Lee, Pyeung-Hyeun Kim, Eui-Bae Jeung, Geun-Shik Lee

**Affiliations:** 1 College of Veterinary Medicine and Institute of Veterinary Science; 2 Department of Molecular Bioscience, School of Biomedical Science, Kangwon National University, Chuncheon 24341, Republic of Korea; 3 Lab. of Veterinary Biochemistry and Molecular Biology, College of Veterinary Medicine, Chungbuk National University, Cheongju 28644, Republic of Korea.

**Keywords:** immune modulator, inflammasome, macrophages, interleukin-1β

## Abstract

Inflammatory responses are essential in eliminating harmful substrates from damaged tissue and inducing recovery. Several cytokines participate in and facilitate this response. Certain cytokines such as interleukin (IL)-1β and IL-18 are initially produced in precursor form in response to toll-like receptor (TLR) ligands and undergo maturation by inflammasomes, which are cytosolic multi-protein complexes containing nucleotide-binding oligomerization domain (NOD)-containing protein 2-like receptors (NLRs). Immune modulators targeting inflammasomes have been investigated to control inflammatory diseases such as metabolic syndrome. However, most immune modulators possessing anti-inflammasome properties attenuate production of other cytokines, which are essential for host defense. In this review, we analyzed the effect of anti-inflammasome agents on the production of cytokines which are not regulated by inflammasome and involving in initial immune responses. As a result, the inflammasome inhibitors are put into three categories: non-effector, stimulator, or inhibitor of cytokine production. Even the stimulator of cytokine production ameliorated symptoms resulting from inflammasome activation in mouse models. Thus, we suggest ideal immune modulators targeting inflammasomes in order to enhance cytokine production while inhibiting cytokine maturation.

## Introduction

Inflammasomes, first introduced by Tschopp and his team in 2002, are multiprotein complexes formed by the mechanism in which inflammatory cytokines, particularly interleukin (IL)-1β and IL-18, are converted from inactive precursors into active form and then secreted^[1]^. Inflammasomes are present in the cytoplasm of immune and non-immune cells, such as macrophages, dendritic cells, and neutrophil cells, as well as keratinocytes, epithelial cells, and adipocytes^[2^–^4]^. Inflammasomes are composed of receptors such as nucleotide-binding oligomerization domain (NOD)-containing protein 2-like receptors (NLRs), which recognize pathogen-associated molecular patterns (PAMPs) and danger-associated molecular patterns (DAMPs), precursor of caspase-1, a cysteine protease, and apoptosis-associated speck-like protein (ASC), an adapter protein^[5]^. Inflammasome components are present throughout the cytoplasm in a steady state, after which they aggregate to form a complex in response to a particular stimulus, which is a process called inflammasome activation. Upon inflammasome activation, pro-caspase 1 self-cleaves to form active hetero-tetrameric caspase 1, which leads to maturation and secretion of pro-inflammatory cytokines. Recent studies suggest that the non-canonical inflammasome detects cytoplasmic lipopolysaccharides (LPS) and activates caspase-11 in mouse macrophages^[6]^. Active caspases enzymatically cleave gasdermin D which forms a hole in the cell membrane and destroys it to induce pyroptosis^[7^–^8]^.

## Priming and activation steps of inflammasome activation

**Fig.1:**
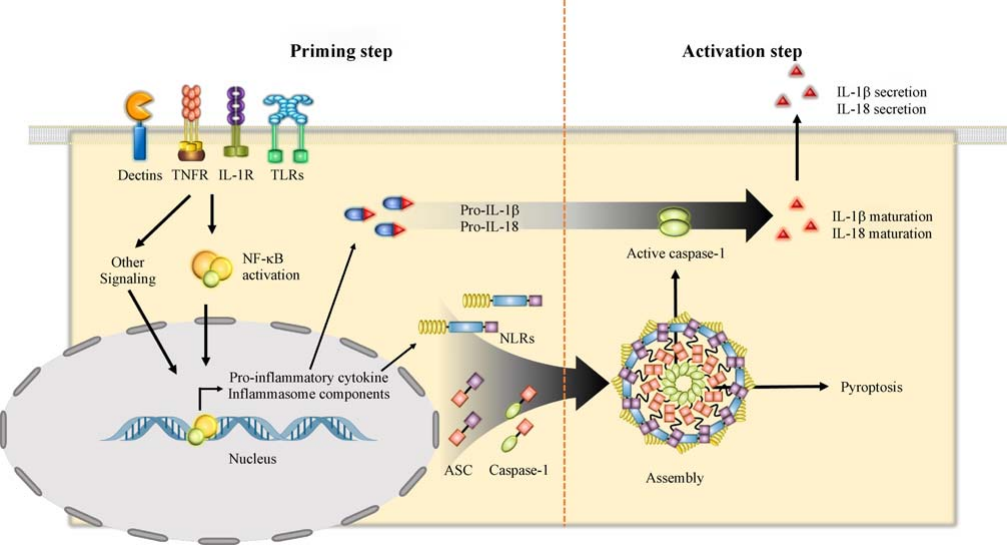
Priming and activation steps.

IL-1β is induced at the transcriptional level upon stimulation with pathogen-derived substances, which mainly interact with toll-like receptors (TLRs), and is translated as a precursor in the cytoplasm, after which it is secreted out of cells after being converted into active form through proteolytic maturation resulting from inflammasome assembly^[9]^. These separate processes are strictly regulated by two checkpoint mechanisms in the process of inflammasome activation^[10]^. Macrophages show no or minimal response to inflammasome activator stimulation, whereas pretreatment with bacterial ligands such as LPS strongly enhances inflammasome activation. Unlike ASC or caspase-1, inflamm-atory cytokines and some inflammasome constitutive proteins such as NLRs are insufficient for activation in dormant macrophages and are expressed upon activation of nuclear factor kappa-light-chain-enhancer of activated B cells (NF-κB) signaling and its downstream kinases through receptors such as TLRs^[11]^. Preparation for inflammasome activation is the first signal, defined as the "priming step" (***Fig. 1***)^[12]^. Inflammasome activity was shown to significantly increase during the priming step for a short period, which was not sufficient to induce expression of inflammatory cytokines and inflammasome components^[12]^. In the NF-kB signaling pathway, myeloid differentiation primary response 88 (MyD88) and its downstream kinases, IL-1 receptor-associate kinase (IRAK)1 and IRAK4, are involved in regulation of transcription levels, whereas IRAK1 and Toll/IL-1 receptor-domain-containing adapter-inducing interferon-β are implicated in the regulation of post-transcription levels^[11]^. In addition, it has been reported that inflammasome activation is regulated through phosphorylation and deubiquitination of inflammasome proteins in the priming step^[13]^. Thus, the role of the priming step is more complicated and is possibly related to fine tuning of inflammasome activation.

After the priming step and stimulation by the inflammasome activator, inflammasome components are assembled to form a complex and caspase-1 activated, which is referred to as the "activation step" (***Fig. 1***)^[14]^. Caspase-1 is a cysteine protease known as IL-1-converting enzyme (ICE), which has excellent activity in converting precursor IL-1β and IL-18 into their mature forms^[15]^. Caspase-1 exists in the cytoplasm in an inactive state with a size of 45 kDa and is cleaved into active fragments (p10 and p20) composed of a heterodimer resulting from inflammasome activation. Precursor caspase-1 (p45) is recruited by interaction with the caspase recruitment domain (CARD) of inflammasome receptor protein or adapter protein (ASC), and auto-proteolysis occurs within the inflammasome. Specifically, the active form of caspase-1 recognizes the Trp-Glu-His-Asp motif and cleaves aspartic acid residues. ASC, which contains an *N*-terminal pyrin domain (PYD) and *C*-terminal CARD domain, plays a central role in inflammasome activation and provides a link between intracellular sensor and caspase-1 proteins^[16]^. Thus, the assembly of inflammasome receptor protein, ASC, and caspase-1 is defined as the activation step resulting in cytokine maturation by heterodimer (p10 and p20) of casaspe-1.

## Inflammasome-related diseases

### Autoinflammatory diseases

Besides autoimmune diseases, “autoinflammatory diseases **”** are classified as diseases in which systemic inflammation frequently reoccurs in the absence of autoantibodies or antigen-specific T cells^[17]^. In addition, autoimmune diseases are often caused by malfunctions in the adaptive immune system, whereas autoinflammatory diseases are caused by malfunctions in the innate immune system. Autoinflammatory diseases are caused by genetic mutations in molecules that are involved in regulating the innate immune response, resulting in excessive secretion of inflammatory cytokines, mainly IL-1, even in the absence of specific immunoreactivity factors such as infectious agents. Various systemic symptoms of autoinflammatory diseases include high fever, rash, arthralgia, chest pain, and abdominal pain^[18]^. Based upon underlying disease-inducing molecular mechanisms, the main cause of autoinflammatory diseases is abnormal inflammasome activation.

### Metabolic diseases

Diabetes was reported to be associated with NLR family PYD-containing protein (NLRP) 3 inflammasome activation, as it causes insulin resistance as well as obesity-associated inflammation^[19]^. Macrophages infiltrate into adipose tissue under obese conditions, and inflammatory mediators released from macrophages cause insulin resistance. Treatment of diabetic patients with IL-1 inhibitors has been shown to inhibit hyperglycemia^[20^–^21]^. Atherosclerosis is a chronic disease resulting in progressive narrowing of arterial vessels due to an imbalance in lipid metabolism, and it was reported that the NLRP3 inflammasome is involved^[22]^. In peripheral blood mononuclear cells (PBMCs) of patients, the gene expressions of NLRP3, caspase-1, and IL-1β are enhanced. Cholesterol crystals in vascular plaques of atherosclerosis are important activators of the NLRP3 inflammasome and have been shown to prevent atherosclerosis in *NLRP3*-deficient mice. In addition, the NLRP3 inflammasome plays an important role in renal disease and hypertension^[23]^. Gout is a disease caused by excessive accumulation of uric acid, which is a metabolite of purine produced by degradation of nuclear proteins in body cells or decomposition of nuclear proteins in ingested food^[24]^. Monosodium urate crystals (MSU) accumulated in joints is recognized by the NLRP3 inflammasome and causes IL-1β cytokine production^[25]^.

### Other inflammasome-related diseases

Inflammatory bowel disease (IBD) is a group of inflammatory conditions affecting the colon and small intestine. Crohn's disease (CD) and ulcerative colitis (UC) are the principal types of IBD. The presence of single-nucleotide polymorphisms (SNPs) in the regulatory regions of the NLRP3 gene and CARD8 gene (which encodes a protein belonging to the CARD) is associated with susceptibility to CD^[26]^. A recent report confirmed NLRP3 inflammasome activation in CD and UC patients with long-standing diseases using an *ex vivo* experimental model of IL-1β level measurement in PBMCs from CD or UC patients compared to the control^[27]^. Conversely, IL-18 production through the NLRP3 inflammatory pathway has been reported to have a protective effect against colitis^[3]^. Alzheimer's disease (AD) is a chronic neurodegenerative disease caused by the accumulation of amyloid-β plaques in the cerebrum. Amyloid-β protein acts to potently activate the NLRP3 inflammasome in microglia, which are the tissue-resident macrophages of the central nervous system^[28]^. The levels of activated caspase-1 and IL-1β in the brain were shown to be increased in AD patients and AD animal models^[29]^. Consistent with these results, age-related astrocyte proliferation, microglia activation, and IL-1 and tumor necrosis factor (TNF) α expression were found to be suppressed in NLRP3-deficient mice. In conclusion, the NLRP3 inflammasome plays an important role in the development of neuroinflammation, and it is involved in the deterioration of cognitive function due to aging. Therefore, identification of the NLRP3-caspase-1 axis is a potential target for AD therapy.

## Treatment of inflammasome-related diseases

### Treatments targeting IL-1 and its receptor

Autoinflammatory diseases are caused by excessive production of inflammatory cytokines due to activation of the innate immune system. Since elevation of IL-1β and IL-18 expression *via* inflammasome activation is the cause of autoinflammatory disease, drugs targeting IL-1 receptor are commonly used^[9]^. Anakinra is a recombinant IL-1 receptor antagonist and differs from native IL-1RA in that methionine is added to the N-terminus. Anakinra blocks the biological activity of naturally occurring IL-1 by competitively inhibiting IL-1 binding to IL-1 receptors expressed in many tissues and organs. Canakinumab is a human monoclonal antibody that targets and has a strong affinity for IL-1β. Rilonacept, also known as IL-1 Trap, is an interleukin 1 inhibitor and dimeric fusion protein consisting of the ligand-binding domains of extracellular portions of human interleukin-1 receptor component and IL-1 receptor accessory protein linked in-line to the fragment-crystallizable portion of human IgG1, which binds to and neutralizes IL-1.

### Treatments targeting inflammasome activation

There are several drugs that directly or indirectly target inflammasomes by regulating production of IL-1. Glyburide, also known as glibenclamide, is the first compound reported to inhibit NLRP3-dependent IL-1β production, whereas it has no effect on NLRP1- or NLR family CARD-containing protein (NLRC) 4-dependent IL-1β secretion^[30]^. Glyburide interacts with P2X7 receptor and NLRP3. Nucleic acid reverse transcriptase inhibitors are also members of the NLRP3 inhibitor group that targets the P2X7 signaling system^[31]^. MCC950, a diarylsulfonylurea-containing compound, is a potent, selective, and orally available inflammasome inhibitor^[32]^. MCC950 blocks release of IL-1β in macrophages primed with LPS and activated by NLRP3 inflammasome activators, but it does not inhibit NLRP1, NLRC4, absent in melanoma 2 (AIM2), or TLR2 signaling or priming of NLRP3. β-Hydroxybutyrate (BHB), a ketone that acts as an alternative source of ATP in an energy-deficit state, also specifically inhibits various stimuli that activate the NLRP3 inflammasome^[33]^. Cholesterol 25-hydroxylase (Ch25h), a substance produced by interferon stimulation, has a broad inhibitory capacity for various inflammasomes^[34]^. Type I interferon has been shown to inhibit inflammasome activation through a currently unknown mechanism. More importantly, 25-hydroxycholesterol, which is the substrate for Ch25h, may inhibit NLRP3 inflammasome activation and IL-1β production.

## Immune modulators

The therapeutic agent used in immunotherapy is called an “immune modulator”, which is a therapeutic agent that either inhibits elevation of the immune response or enhances inhibition of the immune response with no effect on the normal immune response^[35]^. Establishment of the immune system is extremely complicated, and there is no immune modulator that has been completely clarified. Immune modulators require three conditions: absence of antigenicity besides existing pharmacological actions, absence of haptenicity, and absence of polyclonal B cell activation. Immune modulators are a new category of drugs used to treat certain types of immune and inflammatory diseases and are based on a diverse array of recombinant, synthetic, and natural preparations. Recent immunotherapy has attracted great interest of researchers, clinicians, and pharmaceutical companies, especially for the treatment of various forms of cancer. Thousands of patents are filed each year on this subject, and there are estimated thousands of immunotherapy licensing opportunities at technology transfer offices that require development and commercialization partners. Immunomodulatory regimens are less likely to produce resistance when treating microbial diseases and thus have less side effects than traditional drugs. Therapeutic methods such as granulocyte colony-stimulating factor (G-CSF), interferons, imiquimod, and cellular membrane fractions from bacteria are licensed for medical use^[36]^. Others such as IL-2, IL-7, IL-12, various chemokines, synthetic cytosine phosphate-guanosine (CpG) oligodeoxynucleotides, and glucans are currently the subjects of clinical and preclinical studies^[37]^. Immune modulators are becoming a viable adjunct to established modalities, offering a novel approach for the treatment of diseases.

The applicability of inflammasome regulators to the treatment of inflammasome-related diseases, which are mostly chronic wasting diseases such as diabetes, arthritis, gout, AD, and arteriosclerosis, has been investigated. Long-term oral administration of an immune regulator should be less disruptive to host inflammatory responses to infection. For safety, inflammasome regulators in foods or plant extracts should be safe and edible. Newly discovered substances or created compounds are a concern for human safety, but food-derived substances or herbal medicines have long been used in the private sector and consumed as dietary supplements. These substances have no restrictions on their duration and dosage and are advantageous in terms of their side effects and safety. To avoid a host inflammatory response, candidates have been independently tested at the inflammasome priming and activation steps. Expression patterns of cytokines at the priming step were investigated since this step is the same as those in the inflammation response. Taken together, the ideal immune modulator targeting inflammasomes should inhibit the activation step of inflammasomes but not suppress the inflammation response during the priming step.

## Immune modulators targeting inflammasomes

### Dietary sulfur compounds

Methylsulfonylmethane (MSM) is a naturally occurring organosulfur compound with various anti-cancer, antioxidant, and anti-inflammatory properties, and it is utilized in complementary and alternative medicine. MSM obstructs the mRNA transcription of *IL-1* α*, IL-1* β*, IL-6,* and *NLRP3* and inhibits NLRP3 inflammasome activation, thereby reducing IL-1β maturation, active caspase-1 secretion, and ASC pyroptosome formation in macrophages^[38]^. However, MSM has no effect on NLRC4 or AIM2 inflammasome activation, demonstrating selective NLRP3 inflammasome inhibitory effects. Dimethyl sulfoxide (DMSO), the parent compound of MSM, is an amphipathic molecule widely used as a solvent as well as a treatment for various inflammatory diseases. DMSO attenuates gene transcription of the inflammatory cytokines *IL-1* α*, IL-1* β*,* and *IL-6* and suppresses NLRP3 inflammasome activation but not NLRC4 or AIM2 inflammasome activation. MSM and DMSO inhibit production of reactive oxygen species (ROS) and reduce NLRP3 inflammation activation through mitochondrial damage^[39]^. In a prior study, DMSO increased the survival rate in an LPS-induced endotoxemia mouse model and reduced weight loss in a DSS-induced inflammatory bowel disease mouse model.

Sulforaphane (SFN) is a phytochemical (plant-derived substance that does not generally act as a nutrient but exhibits physiological activity) containing sulfur, which is abundant in cruciferous plants such as broccoli. SFN blocks expression of inflammatory cytokines and the NLRP3 gene by TLR2, TLR4, or TLR5 agonists as well as inhibits activation of the NLRP3 and NLRC4 inflammasomes but not AIM2 inflammasome^[40]^. In an MSU-induced peritonitis mouse model, SFN reduced the number of peritoneal exudate cells (PECs) and IL-1β secretion in the peritoneal fluid and serum. In addition, extracts of garlic, onion, chive, and wild chive containing sulfur compounds were found to inhibit NLRP3 inflammasome activation^[38]^. Overall, these sulfur compounds selectively attenuated inflammasome activation and cytokine expression at the priming step. Thus, sulfur compounds are not ideal immune modulators targeting inflammasomes since they suppress both the priming and activation steps of inflammasome activation.

### Hyperoside and isorhamnetin derived from water dropwort

Water dropwort is a perennial herb with a unique fragrance and taste, and it is rich in fiber and minerals such as vitamin A, B1, B2, C, iron, calcium, and phosphorus. In Oriental medicine, water dropwort is believed to have detoxifying, blood cleansing, and blood pressure lowering effects. Extracts from water dropwort inhibit NLRP3, NLRC4, and AIM2 inflammasome activation^[41]^. Hyperoside and isorhamnetin, which are the medicinal components of water dropwort, are quercetin glycosides and flavonoids, which is a subpopulation of polyphenols. Hyperoside is quercetin 3-galactoside while isorhamnetin is a methylated metabolite of quercetin, also known as 3'-O-methyl quercetin. Hyperoside has no effect on cytokine expression but selectively interrupts NLRC4 and AIM2 inflammasome activation. On the other hand, isorhamnetin reduces the expression of inflammatory cytokines such as IL-1β, TNFα, and IL-6 as well as the NLRP3 gene and disturbs NLRP3 and AIM2 inflammasome activation. Thus, hyperoside may be an ideal immune modulator targeting inflammasomes since it selectively attenuates inflammasomes but not inflammatory cytokine transcription. However, isorhamnetin presents similar properties as sulfur compounds, which inhibit both the priming and activation steps of inflammasomes.

### Methylene blue

Methylene blue (MB) is a blue aromatic compound that is often used as a staining agent for the observation of cells or bacteria or as a disinfectant or analgesic for some diseases. MB blocks LPS-mediated production of IL-1β and NLPR3 and reduces gene expression of *IL-1*α*, IL-1*β*, IL-6, IL-10, IL-12b,* and *TNF*α, thereby preventing the priming step of inflammasome activation^[42]^. MB inhibits both the activation of canonical inflammasomes such as NLRP3, NLRC4, and AIM2 as well as non-canonical inflammasomes. Furthermore, it attenuates maturation of IL-1β and IL-18, secretion of active caspase-1, and formation of the ASC pyroptosome. MB inhibits ROS production and phagocytosis of mitochondria in macrophages as well as caspase-1 activity. Furthermore, it blocks NF-κB signaling through the NF-κB-binding site of NLRP3 promoter, which regulates NLRP3 gene expression. MB was also shown to reduce the lethality rate in an LPS endotoxemia model as well as peritoneal IL-1β secretion in a peritonitis model. Thus, MB is not an ideal immune modulator targeting inflammasomes since it inhibits both the production and maturation of inflammatory cytokines.

### Korean Red Ginseng extract

Korean Red Ginseng is an Oriental medicinal herb made from repeated steaming and drying of ginseng. As a popular health food, it is believed to boost immunity and increase energy. Korean Red Ginseng contains various active ingredients, including ginsenosides, polysaccharides, peptides, and fatty acids^[43]^. Ginsenosides, a ginseng saponin, are a representative physiologically active ingredient and have been widely studied in various fields. Especially, the anti-inflammatory and anti-inflammasome activation effects of ginsenosides have been studied^[43^–^44]^. Korean Red Ginseng extract (RGE) and its ginsenosides (Rh1 and Rg3) inhibit activation of the NLRP3 and AIM2 inflammasomes and reduce IL-1β secretion. It was previously confirmed that RGE improved mortality in an LPS endotoxemia model. Similar to previous reports that ginsenosides inhibit NF- κB signaling and reduce inflammatory cytokine production, the saponin fraction (SF) of RGE was shown to inhibit expression of inflammatory cytokines and NLRP3. In contrast to SF, the non-saponin fraction (NS) of RGE induced expression of IL-1β and NLRP3 *via* TLR4^[45]^. In addition, NS did not participate in inflammasome activation while SF showed an inhibitory effect. It can be inferred that the opposite activities of NS and SF on inflammasome activation are active in RGE based on the increased expression of inflammatory cytokines with an inverse concentration gradient of RGE. Therefore, RGE is an ideal immune modulator targeting inflammasomes since the SF attenuates the activation step of inflammasomes while NS stimulates inflammatory responses.

### Poly-γ-glutamic acid

Poly-γ-glutamic acid (PGA) is a metabolite produced by fermentation of soybeans using *Bacillus* species. PGA is a non-toxic, water-soluble, and biodegradable macromolecule compound that has been reported to exhibit fibrin degradation, anti-cancer, anti-inflammatory, and antioxidant effects^[46^–^47]^. PGA enhances immune function by inducing cytokine secretion *via* TLR4-mediated signaling, activates natural killer (NK) cells by interferon-β and IL-12 secretion, and promotes interferon-γ secretion in T cells^[47]^. PGA has also been reported to activate immune cells such as NK cells as well as inhibit intracellular bacterial and viral infections and cancer cell proliferation^[48]^. PGA suppresses both the activation of canonical inflammasomes such as NLRP3, NLRC4, and AIM2 and non-canonical inflammasomes^[46]^. PGA has the contradictory effect of increasing gene expression of IL-1β, an inflammatory cytokine, and reducing protein maturation levels, although it was found to reduce the lethality rate in an LPS endotoxemia model. Taken together, PGA shows the best potential as an immune modulator targeting inflammasomes since it attenuates inflammasome activation but increases the inflammatory response with high safety in endotoxemic mice.

### Lentinan

Lentinan (LNT), or β-glucan, is a naturally occurring polysaccharide in the cell walls of cereals, bacteria, and fungi that shows significantly differing physicochemical properties depending on the source. It has been reported that β-glucans activate immune cells such as macrophages and promote production of interferon. In particular, LNT stimulates the receptor dectin-1 to induce activation of immune cells, which makes it easy to find and attack viruses that have penetrated into the body^[49]^. The type of β-glucan extracted from shiitake mushroom LNT, which is known to have anti-cancer effects, enhances expression of inflammatory cytokines and inflammasome components as well as reduces activation of the AIM2 and non-canonical inflammasomes^[50^–^51]^. In a peritonitis model of *Listeria monocytogenes* infection that activates the AIM2 inflamma-some, LNT reduced secretion of IL-1β in the abdominal cavity and diminished the mortality rate in an LPS endotoxemia model. Similar to PGA, LNT is an ideal immune modulator targeting inflammasomes without loss of the inflammatory response against bacterial infection.

## Summary

In this review, we screened immune modulators targeting inflammasome activation to control inflammatory-related diseases. We also investigated the effects of the modulators on the priming step of inflammasome activation as a part of the inflammatory response (***Table 1***). All substrates described in this paper showed anti-inflammasome properties and blocked of inflammasome assembly, resulting in inhibition of IL-1β, IL-18, and caspase-1 secretion. However, these substances differed in their effects on the priming step, which plays a role in regulating the transcription levels of inflammatory cytokines that mediate inflammatory responses. MSM, MB, isorhamnetin, and SF of RGE inhibited TLR4-dependent NF-κB signaling by LPS and suppressed expression of inflammatory cytokines and inflammasome components. In contrast, NS of RGE, PGA, and LNT stimulated TLR4 by activating NF-κB signaling, leading to expression of inflammatory cytokines and inflammasome components. However, hyperoside did not alter cytokine expression. Interestingly, substrates inducing TLR4-NF-κB signaling ameliorated symptoms in LPS endotoxemia and various peritonitis animal models. Thus, RGE, LNT, and PGA can be suggested as ideal immune modulators targeting inflammasome activation since they were found to stimulate cytokine production while inhibiting cytokine maturation. That is, these modulators boost the host immune system *via* expression of cytokines but block fatal symptoms by inhibition of inflammasome activation.

**Tab.1:** Immune modulators targeting inflammasomes

Substances				Priming step		Activation step			
						NLRP3	NLRC4	AIM2	Non–canonical
Dietary sulfur compounds									
	Methylsulfonylmethane (MSM)			↓		↓	–	–	Not tested
	Dimethylsulfoxide (DMSO)			↓		↓	–	–	Not tested
	Sulforaphane (SFN)			↓		↓	↓	–	Not tested
Methylene blue (MB)						↓	↓	↓	↓
Water dropwort derivative									
	Hyperoside			–		–	↓	↓	Not tested
	Isorhamnetin			↓		↓	–	↓	Not tested
Korean Red Ginseng extract									
	Saponin fraction (SF)			↓		↓	↓	↓	Not tested
	Non–saponin fraction (NS)			↑		–	–	↓	–
Poly–gamma–glutamic acid (PGA)				↑		↓	↓	↓	↓
Lentinana (LNT)				↑		–	–	↓	↓

↓: inhibition, ↑:Stimulation, –: No–effect

## Conclusion

Inflammasomes are cytoplasmic protein complexes that mediate maturation of the pro-forms of IL-1β and IL-18, which are key cytokines in the inflammatory response. Inflammasome activation can be divided into the priming and activation steps. The priming step increases the expression levels of cytokine precursors and inflammasome components in response to stimulation by TLR ligands. Subsequently, the activation step induces the assembly of inflammasome components, resulting in cleavage of caspase-1 and the pro-forms of cytokines. Various types of inflammasomes have been identified in immune and non-immune cells, and each inflammasome plays a distinct role in inflammation and wound healing. Dysregulation of inflammasomes causes auto-inflammatory diseases such as metabolic syndrome. Thus, immune modulators targeting inflammasome regulation are currently being investigated and developed. However, most researchers have focused on the activation step of inflammasomes such as secretion of IL-1β, which triggers and amplifies systemic inflammation. Activation of inflammasomes cannot start without the priming step, which not only regulates expression of inflammasome components but also alters levels of other inflammatory cytokines. Immune modulators, both stimulators and inhibitors, are commonly defined based on their effects on cytokine expression, as in the priming step, and not based on maturation resulting from inflammasome activation. In this review, we summarized several candidates based on their anti-inflammasome properties and further divided them into types based on their effects on the NLRP3, NLRC4, AIM2, and non-canonical inflammasomes. Furthermore, the effects of the molecules on the priming step of inflammasome activation were analyzed in human and mouse macrophages. The molecules were put into three categories (***Table 2***): stimulator of priming and activation steps (both-up), inhibitor of both steps (both-down), or simulator of priming step while inhibitor of activation step (up/down). The molecules classified as both-up were typical inflammasome triggers such as LPS and MSU. The molecules categorized as both-down were defined as anti-inflammatory agents such as dietary sulfurs. Finally, the other substrates classified as up/down were immune stimulators such as PGA, LNT, and RGE. In an animal study, both-up molecules induced cytokine secretion and death while up/down molecules did not present any symptoms. When both-up and up/down molecules were both applied in mice, the toxic effect of both-up molecules was ameliorated. Thus, the priming step may act as an immune simulator but is not a critical factor for toxicity. The activation step of inflammasomes is a gatekeeper for judging the safety of immune modulators.

**Tab.2:** Classification based on regulation of inflammasome priming and activation

Classification		Definition				Substances		References
Both-up		a stimulator of priming and activation				Lipopolysaccharide (LPS)		^[52^, ^53]^
						Monosodium urate (MSU)		^[54^,^55]^
Both-down		an inhibitor of priming and activation				Methylsulfonylmethane (MSM)		^[38]^
						Dimethylsulfoxide (DMSO)		^[39]^
						Sulforaphane (SFN)		^[40]^
						Isorhamnetin		^[41]^
						Saponin fraction (SF) of Korean Red Ginseng		^[43]^
						Methylene blue (MB)		^[42]^
Up/Down		a stimulator of priming and an inhibitor of activation				Non-saponin fraction (NS) of Korean Red Ginseng		^[45]^
						Lentinana (LNT)		^[50]^
						Poly-gamma-glutamic acid (PGA)		^[46]^
